# Treatment of angiotensin-converting enzyme inhibitor related angioedema with icatibant

**DOI:** 10.1186/1710-1492-10-S2-A18

**Published:** 2014-12-18

**Authors:** Peter Ho, Chrystyna Kalicinsky

**Affiliations:** 1 Section of Allergy and Clinical Immunology, Department of Internal Medicine, University of Manitoba, Winnipeg, Manitoba, MB R3A 1R9, Canada

## Background

The absolute risk of angiotensin-converting enzyme (ACE) inhibitor angioedema is 0.3% [1]. The mechanism is felt to be accumulation of bradykinin. The current treatment is discontinuation of ACE-I inhibitor, and intubation if necessary. Icatibant is a bradykinin receptor antagonist, useful in treating hereditary angioedema [2]. We present a patient from a Canadian center who had ACE-inhibitor induced angioedema requiring intubation who did not respond to epinephrine or C1 esterase inhibitor concentrate, then later responded favorably to Icatibant.

## Case presentation

We present a case of a 76 year old male with hypertension, type II diabetes, no allergies, who was seen in the ER with acute new-onset tongue and facial swelling. He had a virus 2 weeks prior. His medications included quinapril, simvastatin, repaglinide, metformin, pioglitazone, and aspirin.

He had a swollen tongue, oral cavity, neck and was unable to swallow. There was no urticaria. He was given diphenhydramine 50mg IV, methylprednisolone 125mg IV, epinephrine 0.3mg IM, and ranitidine 50mg IV. Swelling progressed and he was intubated.

Over the next 24 hours, he received methylprednisolone 80mg every 8 hours, ranitidine 50mg every 8 hours, diphenhydramine 50mg every 6 hours, two doses of C1 esterase inhibitor concentrate, 1500 units IV followed by 1000 units. Quinipril was discontinued. Computed tomography of the neck showed no abscess.

Over the next 2 days there was minimal improvement. Therefore, on hospital day 3, he received 3 doses of Icatibant 30 mg over 24 hours. Improvement was noted, and by the next day, he was extubated. Following overnight monitoring, he was transferred to the medicine ward. There was no recurrence and he was discharged. C1 esterase inhibitor level and function was normal (drawn prior to infusion).

At his two month follow up he remained asymptomatic. ACE inhibitors and Angiotensin Receptor Blockers are avoided. He was diagnosed with myelodysplastic syndrome following work up for anemia noted on admission.

There have been published reports outside of Canada describing the use of Icatibant in ACE-inhibitor induced angioedema [3].

Icatibant is not indicated in Canada for ACE-inhibitor induced angioedema. However, it may need to be considered in severe cases.

## Conclusion

Icatibant may be helpful in ACE-inhibitor induced angioedema when the patient has not responded to the discontinuation of the ACE inhibitor and alternative therapies.

## Consent

Written informed consent was obtained from the patient for publication of this abstract and any accompanying images. A copy of the written consent is available for review by the Editor of this journal.
